# Multiple Tobacco Product Use among Adolescents with Asthma in Korea

**DOI:** 10.3390/ijerph19159633

**Published:** 2022-08-05

**Authors:** Seokhwan Kim, Kyuhee Jo

**Affiliations:** 1 Department of Health Information, Dongguk University, Wise Campus, 123, Dongdae-ro, Gyeongju-si, Gyeongsangbuk-do 38066, Korea; 2 College of Nursing, Korea University, Anam-Dong, Seongbuk-Gu, Seoul 02841, Korea

**Keywords:** asthma, smoking, multiple tobacco product use

## Abstract

Few studies have examined the use of multiple tobacco products among adolescents with asthma. The purpose of this study was to examine multiple tobacco product use and smoking behaviors. In this study, data from the Korea Youth Risk Behavior Web-based Survey (KYRBWS) were used, and 57,303 samples from 400 middle schools and 400 high schools in Korea were classified as study participants. Statistical analysis was performed with a complex sample design, using frequency analysis, chi-square test, and multiple logistic regression analysis. Adolescents with asthma had a higher current smoking rate for combustible cigarettes (CC), e-cigarettes (EC), and heated tobacco products (HTPs) than those without asthma (*p* < 0.001). The rates of dual use (using two types of tobacco; CC + EC or CC + HTPs or EC + HTPs; OR = 2.62, 95% CI: 1.626–4.240, R^2^ = 26.8%) and triple use (using three types of tobacco; CC + EC + HTPs; OR = 2.61, 95% CI: 1.678–4.065, R^2^ = 34.9%) were higher in adolescents with asthma than those without asthma, after adjusting for confounders. The smoking rate of new types of tobacco among adolescents with asthma is on the rise. Therefore, the calculation of basic data related to new tobacco smoking among adolescents is essential for establishing a continuous monitoring system to alleviate the burden of disease on national health.

## 1. Introduction

Asthma is one of the most common non-communicable diseases of all age groups [1] and is a representative allergic disease with airflow restriction symptoms such as dyspnea, wheezing, and coughing [2]. More than 340 million people suffer from asthma worldwide [3,4]. In the United States, 8.3% of people have asthma, and 20.4 million out of 26.5 million people are over the age of 18 have it, while 6.1 million are under the age of 18 [5]. In Korea, asthma has the sixth highest disease burden among chronic diseases, and the hospitalization rate for asthma is twice the Organization for Economic Co-operation and Development (OECD) average [6]. The prevalence of asthma among Korean adolescents is increasing every year, and the diagnosis rate of asthma doctors in Korea in 2020 was 6.2% [7]. In Korea, asthma tends to occur more frequently in children and adolescents than in adults [8].

Smoking is a risk factor for many adverse effects of asthma [9,10], and diseases caused by smoking can be prevented by cessation of smoking [11]. In 2020, in the United States, 16.2% (4.47 million) of middle and high school students, 23.6% (3.65 million) of high school students, and 6.7% (800,000) of middle school students smoked at the time of the survey [12]. The current smoking rate of adolescents in Korea decreased by 6.6% (from 13.3% in 2007 to 6.7% in 2019), and the current smoking rate of high school students (9.9%) was 3.1 times higher than that of middle school students (3.2%) [7]. In addition, as of 2019, the current smoking rates of e-cigarettes and heated tobacco products (HTPs) among Korean adolescents were 3.2% and 2.6%, respectively [13]. Smoking among adolescents with asthma lowers lung function and adversely affects chronic diseases [14].

Approximately 12% of Korean adolescents are smokers, and effective management is required because smoking and secondhand smoking can increase the incidence of asthma in adolescents [11]. In addition, the use of e-cigarettes among adolescents is increasing [15], and e-cigarettes use liquid substances, which may or may not contain nicotine. The liquid substances in e-cigarettes contain additives and chemicals that can have long-term health effects [16]. Therefore, the potential risks and benefits of e-cigarettes require further research. E-cigarettes are also known to help smokers reduce their use of traditional combustible cigarettes and are marketed as tobacco substitutes and smoking cessation aids [17,18]. Some adolescents who currently do not smoke using e-cigarettes are at risk of smoking traditional cigarettes [19,20]. Most e-cigarette users among Korean adolescents are dual smokers [13].

Adolescent smokers with asthma showed unhealthy behavior and a tendency for a lowered ability for disease control than those with asthma who did not smoke [21,22]. Therefore, the purpose of this study was to analyze smoking behavior for the use of combustible cigarettes (CC), e-cigarettes (EC), heated tobacco products (HTPs), and multiple tobacco products among adolescents with and without asthma.

## 2. Materials and Methods

### 2.1. Design and Participants

The data for this study were taken from 2015, 2016, 2017, 2018, and 2019 “2019 Korea Youth Risk Behavior Web-based Survey (KYRBWS),” conducted by the Korea Centers for Disease Control and Prevention. The Adolescent Health Behavior Survey is an anonymous, self-reported online survey conducted annually for middle and high school students to understand the health behaviors of Korean adolescents, such as smoking, drinking, physical activity, and eating habits. The 15th survey in 2019 was conducted with 60,100 students from 400 middle and 400 high schools, with a response rate of 95.3% based on the number of students. All participants were included in calculating the smoking rate and asthma prevalence among adolescents, except for some missing values without missing samples from the raw data for each order.

### 2.2. Measurements

This study used the 2019 KYRBWS data for sociodemographic, behavioral, and smoking behavior variables. Sociodemographic variables included gender (boys and girls), age, school (middle school or high school), family economic level (good, fair, or bad), and living with family (yes or no). Behavioral variables included current drinking (yes or no), regular exercise (low or high), and BMI. BMI is weight divided by height squared. Weight and height were calculated using self-reporting measures. Regular exercise was recorded as high if moderate-intensity or higher physical activity was performed three or more days per week, as per the American physical activity guidelines [23]. Smoking behavior variables were exposure to secondhand smoke and cigarette pack warning pictures. Current asthma was defined as a case of having an asthma diagnosis made by a physician within the last 12 months. Current smoking was defined as smoking for at least one day within the last 30 days. Regarding current smoking status, respondents who answered yes to the following questions were classified as current smoking. “Have you smoked combustible cigarettes, liquid e-cigarettes containing nicotine, and heated tobacco products (e. g. IQOS, glo, lil) in the last 30 days? (each).”

### 2.3. Ethical Consideration

This study was approved by the Institutional Review Board at Korea National Institute for Bioethics Policy for the ethical aspects related to the use of research data, the research plan, and the pledge of research ethics (approval number: P01-202008-22-003).

### 2.4. Data Analysis

In this study, SPSS 23.0 (IBM, Chicago, IL, USA) was used for analysis. As a cross-sectional study, this study aimed to understand the disease patterns and related attributes of the entire target group and to compare the complex smoking behavior of people with and without asthma. The statistical analysis method was as follows. First, the frequency of asthma prevalence and smoking rate among Korean adolescents with asthma for five years was analyzed for all study participants by year. Second, the weights were stratified, and a complex sample design was applied to calculate the asthma prevalence and recent smoking rate among adolescents in Korea. The smoking rate and complex smoking behavior for each cigarette (combustible cigarette + e-cigarette, combustible cigarette + heated tobacco products, e-cigarette + heated tobacco products, combustible tobacco + e-cigarette + heated tobacco products) smoking rate and five-year increase/decrease values were derived. Third, statistically significant differences in potential risk factors, such as socioeconomic factors and smoking-related factors, were verified between the groups of adolescent smokers with and without asthma through the Rao–Scott chi-square test. Lastly, the relationship between the complex smoking-related asthma and non-asthma groups was identified using logistic regression analysis.

## 3. Results

### 3.1. Rate of Cigarettes Use by Asthma Status

**Figure 1:**
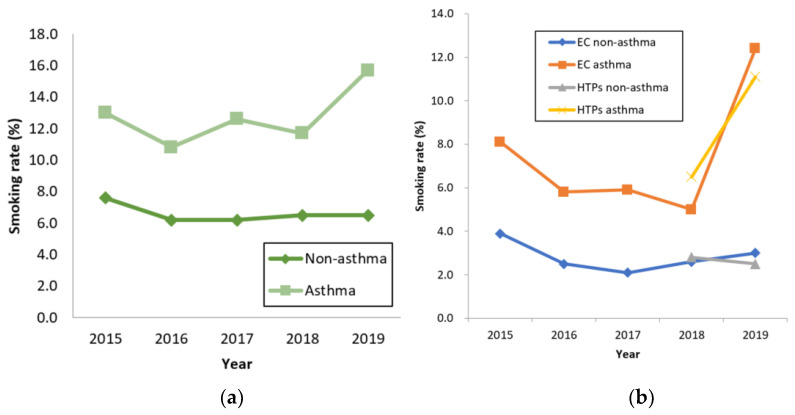
Rate of cigarette use by asthma status: (**a**) rates of combustible cigarette use by asthma status; (**b**) rates of e-cigarette and heated tobacco product use by asthma status.

In Korea, although the smoking rate of non-asthma has hardly changed, the smoking rate of asthma is increasing (Figure 1a). In 2019, the smoking rates of e-cigarettes and heated tobacco products among asthmatic smokers in Korea were about 4.1 and 4.4 times higher than that of non-asthma smokers (Figure 1b).

### 3.2. Participants’ General Characteristics

In the study, 1.66% (44,518/2,683,547) was diagnosed with asthma during the past 12 months. The proportion of current asthmatic boys was 58.2% (25,904/44,518). Compared to the non-asthma group, the adolescents with the asthma group were 2.4 times more likely to smoke combustible cigarettes, and with e-cigarettes they were approximately 4.1 times more likely (Table 1).

### 3.3. Difference in Smoking Behaviors by Asthma Status

Approximately 35% of smokers with asthma used e-cigarettes or HTPs. On the other hand, non-asthma smokers accounted for 10%. Secondhand smoke exposure at school among smokers with asthma was approximately 60%, and that of without asthma was 35%. Cigarette pack warning picture recognition rate was 16% among smokers with asthma and 10% in those without asthma (Table 2).

### 3.4. Multiple Tobacco Product Use by Asthma Status

The multiple tobacco product use rates among smokers with and without asthma were approximately 40% and 22%, respectively (Table 3).

### 3.5. The Association of Asthma Status with Multiple Tobacco Product Use

Compared to smokers without asthma, the odds ratio for multiple tobacco product use was found to be 2.6 times for smokers with asthma (Table 4).

## 4. Discussion

This study investigated the association between asthma and multiple tobacco use in a nationwide sample of adolescents in Korea. Compared to non-asthmatics, adolescents with asthma use combustible cigarettes (CC), e-cigarettes (EC), heated tobacco products (HTPs), and use multiple tobacco products more. These findings pointed out the association between asthma and current smoking.

In this study, the prevalence of asthma in 2019 was 7.2%, and 1.66% (44,518/2,683,547) were diagnosed with asthma during the past 12 months. The smoking rate of asthmatic adolescents was higher than that of the non-asthmatic group. This result was consistent with previous studies [23,24,25,26]. Several studies reported that active smokers with asthma have more prevalent comorbidity, including lung cancer [27], depression, anxiety [28], and cardiovascular disease [29]. Similarly, several recent studies reported a high odds ratio with asthma in current e-cigarette smokers who had never actively smoked [10,30,31]. According to a study of Australia, adolescents with asthma smoked 2.55 times more, daily, than general middle and high school students. The results of this study also showed that adolescents with asthma were twice as likely to smoke than those without asthma [32].

With the advent of e-cigarettes, adolescent smoking rates among nonsmokers have increased, and even adolescent smokers use e-cigarettes [33,34,35]. In this study, asthmatic e-cigarette smokers increased, and non-asthmatic e-cigarette smokers did not decrease. There is growing evidence of the harmful effects of e-cigarettes, including nicotine [36], e-cigarette use may worsen lung function [37], and e-cigarette smokers have a higher odds ratio for missing class than nonsmokers [38]. According to a study of 2086 adolescents in Southern California, e-cigarette users were twice as likely to suffer from bronchitis as nonsmokers [30]. E-cigarette use in adolescents may affect asthma exacerbation [39].

People with asthma smoked more HTPs than the non-asthmatic group. The smokers with asthma in this study also showed a high rate of smoking HTPs and an even higher rate of multiple tobacco product use [40,41].

Recently, the youth perceived that HTPs are less harmful to the human body than combustible tobacco and tend to accept new types of tobacco easily because they produce less smoke [42]. Their use rate has more than doubled [43], and HTPs release nicotine and other harmful chemicals [44,45]. Analysis of the emissions of heated tobacco products detected 84% of nicotine as compared to combustible tobacco, and HTPs are also related to nicotine addiction [44]. According to the results of this study, asthma smokers were approximately twice as likely to smoke, including HTPs, as compared to smokers without asthma.

The recent rapid increase in the smoking rate of HTPs requires education and health authorities to recognize that they are yet another type of smoking. In the education program, HTPs should be emphasized as containing equally harmful substances for the human body as combustible cigarettes and are not actually smoking cessation aids. In fact, the WHO recommends that smoking cessation policymakers in each country specify that HTPs have the same adverse health effects as combustible tobacco [46]. Due to the increase in smoking, asthma in adolescents is transferred as respiratory diseases in adulthood [34]. It can pose a tremendous financial threat to the public healthcare system.

Several studies describe multiple tobacco use [41,47]. According to recent literature, multiple tobacco use was prevalent among adolescents. Multiple tobacco product use among adolescents may promote and reinforce nicotine addiction. Moreover, they are also more likely to fail smoking cessation, excessive gambling, illicit drug use, and heavy drinking [48]. Our study examined the single-use, double-use, and triple-use tobacco use, and people with asthma smoked multiple tobacco use than the non-asthmatic group. Of current asthmatic smokers, about 40% were triple users (CC + EC + HTPs).

People with asthma should not smoke [49]. Smoking cessation is the most urgent and critical management strategy for adolescents with asthma [50]. Healthcare providers must understand the frequency and intensity of multiple tobacco use in asthmatic youth. An in-depth assessment of smoking behavior and personalized advice are needed to quit smoking [49]. Through this process, symptoms can be improved, and asthma-related quality of life and lung function can be improved—the basis for understanding the transition of asthma to prognosis and respiratory diseases [51]. Advice to quit smoking should be carried out simultaneously in schools, hospitals, and communities. Strict regulation of youth tobacco use is needed.

In general, although warnings on cigarette packs reduce smoking rates and have had a positive effect on smoking cessation success [52], the results of this study showed that smokers with asthma had an insensitive response to cigarette pack warnings compared to smokers without asthma. People with relatively high socioeconomic status tend to react sensitively to warning phrases on cigarette packs [53]. Many adolescents with asthma are more likely to have unhealthy behaviors because they are raised in low socioeconomic status and can be easily exposed to smoking environments [54]. Smoking is a significant variable that worsens asthma [55]; therefore, smoking cessation education for adolescents diagnosed with asthma is extremely important.

Asthma in adolescents not only lowers academic ability but is also a crucial factor in school absences [56]. Among adolescents with asthma in California, USA, asthma was associated with higher school absenteeism [57]. In the case of Korean adolescents in 2017, it was found that 19.4% of students with doctor-diagnosed asthma had been absent for the past 12 months [58]. Asthma management through the prevention of direct and secondhand smoking among adolescents is expected to not only improve health [59] but also contribute to the overall quality of life, such as lowering absenteeism.

This study has several limitations. First, since the data used in the study were cross-sectional causal inferences, the association between smoking and compound smoking in adolescents with asthma cannot be guaranteed. Therefore, longitudinal studies are required to establish causal relationships. Second, the current smoking status variable used in this study may have been underestimated (or overestimated) compared to the actual smoking rate because the results were obtained based on the respondent’s memory of whether or not they smoked in the last 30 days. Third, the results of this study cannot be generalized to other ethnic groups or developing countries.

## 5. Conclusions

This study analyzed factors related to multiple tobacco product use among adolescents with asthma based on national data from the Korea Youth Risk Behavior Web-based Survey (KYRBWS) conducted by the Korea Centers for Disease Control and Prevention. Through this study, it was possible to observe how the usage rates of nicotine-containing e-cigarettes and HTPs among Korean adolescents with and without asthma have changed over the past five years. It was also possible to analyze the change in the asthma compound smoking rate, which was used at the same time each year. Smokers with asthma in Korea showed detrimental smoking behavior and higher multiple tobacco product use rates than those without asthma. Based on the results of this study, we intend to provide basic data for preemptive health management and smoking cessation education on the use of multiple tobacco products among adolescents with asthma.

## Figures and Tables

**Table 1:** General characteristics of study population.

	Non-Asthmatics				Asthmatics				t/χ^2^	*p*-Value
	Unweighted		Weighted		Unweighted		Weighted			
	N	%	N	%	N	%	N	%		
Sex										
Boy	29,301	52.0	1,368,614	51.9	540	57.4	25,904	58.2	15.080	<0.001
Girl	27,062	48.0	1,270,415	48.1	400	42.6	18,614	41.8		
Age	15.08 ± 0.02				14.96 ± 0.65				653.730	<0.001
BMI	21.34 ± 0.28				22.18 ± 0.13				770.190	<0.001
School										
High	27,489	48.8	1,377,943	52.2	430	45.7	21,385	48.0	6.531	0.021
Middle	28,874	51.2	1,261,085	47.8	510	54.3	23,134	52.0		
SES										
Good	22,105	39.2	1,046,105	39.6	400	42.6	19,313	43.4	20.243	<0.001
Fair	27,070	48.0	1,263,496	47.9	387	41.2	18,221	40.9		
Bad	7188	12.8	329,427	12.5	153	16.3	6985	15.7		
Living with family										
With family	53,425	94.8	2,520,841	95.5	842	89.6	40,237	90.4	43.772	<0.001
Without family	2938	5.2	118,186	4.5	98	10.4	4282	9.6		
Regular exercise										
Low	37,867	68.1	1,798,043	67.2	567	62.0	27,583	60.3	15.910	<0.001
High	18,496	31.9	840,985	32.8	373	38.0	16,935	39.7		
Current drink										
No	48,187	85.5	2,247,373	85.2	716	76.2	33,632	75.5	59.011	<0.001
Yes	8176	14.5	391,656	14.8	224	23.8	10,887	24.5		
Current CC										
No	52,807	93.7	2,466,414	93.5	803	85.4	37,540	84.3	93.452	<0.001
Yes	3556	6.3	172,614	6.5	137	14.6	6978	15.7		
Current EC										
No	54,752	97.1	2,559,804	97.0	833	88.6	38,988	87.6	160.615	<0.001
Yes	1611	2.9	79,224	3.0	107	11.4	5531	12.4		
Current HTPs										
No	55,064	97.7	2,573,718	97.5	843	89.7	39,560	88.9	156.571	<0.001
Yes	1299	2.3	65,310	2.5	97	10.3	4959	11.1		

Note. BMI: body mass index; SES: socioeconomic status; CC: combustible cigarettes; EC: e-cigarettes; HTPs: heated tobacco products.

**Table 2:** Difference in smoking behaviors by asthma status.

	Non-Asthmatics				Asthmatics				t/χ^2^	*p*-Value
	Unweighted		Weighted		Unweighted		Weighted			
	N	%	N	%	N	%	N	%		
Quit smoking trials (for one year)										
No	1226	31.8	59,790	31.8	53	31.7	2640	31.5	4192.504	<0.001
Yes	2635	68.2	128,168	68.2	114	68.3	5732	68.5		
Daily use (in the last 30 days)										
CC No	2047	55.7	98,484	55.0	60	43.2	3030	42.6	8.656	0.004
CC Yes	1631	44.3	80,471	45.0	79	56.8	4077	57.4		
EC No	2298	89.0	113,292	88.8	84	64.6	4514	65.8	47.820	<0.001
EC Yes	284	11.0	14,238	11.2	46	35.4	2350	34.2		
HTPs No	1712	98.7	85,790	87.8	73	61.9	3849	63.4	43.504	<0.001
HTPs Yes	23	1.3	11,916	12.2	45	38.1	2224	36.6		
Secondhand home exposure in past 7 days										
No	2192	56.77	107,774	57.3	58	34.7	3029	36.2	29.742	<0.001
Yes	1669	43.23	80,184	42.7	109	65.3	5343	63.8		
Secondhand school exposure in past 7 days										
No	2506	64.91	121,135	64.4	66	39.5	3318	39.6	41.628	<0.001
Yes	1355	35.09	66,823	35.6	101	60.5	5054	60.4		
Secondhand public exposure in past 7 days										
No	1369	35.46	65,744	35.0	38	22.8	1830	21.9	13.480	<0.001
Yes	2492	64.54	122,214	65.0	129	77.2	6542	78.1		
Cigarette pack warning picture awareness										
Yes	3459	89.59	168,875	89.8	138	82.6	6999	83.6	6.013	0.013
No	402	10.41	19,083	10.2	29	17.4	1373	16.4		
Cigarette pack warning picture health risk recognition										
Yes	2961	85.60	143,883	85.2	110	79.7	5591	79.9	2.812	0.071
No	498	14.40	24,992	14.8	28	20.3	1408	20.1		
Attempting to quit smoking by cigarette pack warning										
Yes	2713	78.43	131,837	78.1	108	78.3	5451	77.9	0.003	0.960
No	746	21.57	37,038	21.9	30	21.7	1548	22.1		

Note. CC: combustible cigarettes; EC: e-cigarettes; HTPs: heated tobacco products.

**Table 3:** Multiple tobacco product use by asthma status.

		Non-Asthmatics				Asthmatics				χ^2^	*p*-Value
		Unweighted		Weighted		Unweighted		Weighted			
		N	%	N	%	N	%	N	%		
Single	Only CC	1860	48.2	88,853	47.3	43	25.7	2029	24.2	68.695	<0.001
	Only EC	171	4.4	8540	4.5	7	4.2	303	3.6		
	Only HTPs	52	1.3	2610	1.4	7	4.2	293	3.5		
Dual	CC + EC	531	13.8	25,255	13.4	20	12.0	1081	12.9		
	CC + HTPs	338	8.8	17,271	9.2	10	6.0	518	6.2		
	EC + HTPs	82	2.1	4195	2.2	16	9.6	797	9.5		
Multiple	CC + EC + HTPs	827	21.4	41,234	21.9	64	38.3	3350	40.0		

Note. CC: combustible cigarettes; EC: e-cigarettes; HTPs: heated tobacco products.

**Table 4:** Multiple logistic regression model of the association of asthma status with multiple tobacco product use.

Asthma Status	Single Use		Dual Use		Triple Use	
Asthma	1.607	(1.414–2.264)	2.626	(1.626–4.240)	2.612	(1.678–4.065)
Non-asthma	1		1		1	
	R^2^ = 20.4%		R^2^ = 26.8%		R^2^ = 34.9%	

Note. Adjusted for age, BMI, sex, living with family, family economic status, secondhand smoking (home, school, public), current drinking, regular exercise.
